# JIB-04, A Small Molecule Histone Demethylase Inhibitor, Selectively Targets Colorectal Cancer Stem Cells by Inhibiting the Wnt/β-Catenin Signaling Pathway

**DOI:** 10.1038/s41598-018-24903-0

**Published:** 2018-04-26

**Authors:** Min Seong Kim, Hye In Cho, Hee Jung Yoon, Ye-Hyeon Ahn, Eun Jung Park, Yan Hua Jin, Yeun Kyu Jang

**Affiliations:** 1 Department of Systems Biology, College of Life Science and Biotechnology, Yonsei University, Seoul, 03722 Republic of Korea; 2 Initiative for Biological Function & Systems, Yonsei University, Seoul, 03722 Republic of Korea; 3 Immunotherapeutics Branch, Graduate School of Cancer Science and Policy, National Cancer Center, Goyang, Gyeonggi 10408 South Korea; 4 Institute for Regenerative Medicine, Yanbian University, Yanji, 133002 China; 5 Department of Cell Biology and Genetics, College of Medicine, Yanbian University, Yanji, 133002 China

## Abstract

Although several epigenetic modulating drugs are suggested to target cancer stem cells (CSCs), additional identification of anti-CSC drugs is still necessary. Here we showed that JIB-04, a pan-selective inhibitor of histone demethylase(s), was identified as a small molecule that selectively target colorectal CSCs. Our data showed that JIB-04 is capable of reducing self-renewal and stemness of colorectal CSCs in three different colorectal cancer cell lines. JIB-04 significantly attenuated CSC tumorsphere formation, growth/relapse, invasion, and migration *in vitro*. Furthermore, JIB-04-treated colorectal cancer cells showed reduced tumorigenic activity *in vivo*. RNA sequencing analysis revealed that JIB-04 affected various cancer-related signaling pathways, especially Wnt/β-catenin signaling, which is crucial for the proliferation and maintenance of colorectal cancer cells. qRT-PCR and TOP/FOP flash luciferase assays showed that JIB-04 down-regulated the expression of Wnt/β-catenin-regulated target genes associated with colorectal CSC function. Overall, the effects of JIB-04 were equal to or greater than those of salinomycin, a known anti-colorectal CSC drug, despite the lower concentration of JIB-04 compared with that of salinomycin. Our results strongly suggest that JIB-04 is a promising drug candidate for colorectal cancer therapy.

## Introduction

Colorectal cancer is one of the leading causes of cancer death in developed countries^1^. Despite great efforts to develop more effective therapies in the last decade, clinical trials have shown only partial improvements because of colorectal cancer relapse and recurrence.

Recently, many studies have reported that very small populations of cells, referred to as cancer initiating cells or cancer stem cells (CSCs), in the bulk of colon tumors have self-renewal ability and multi-lineage differentiation potential^2–6^. Similar to intestinal stem cells in normal tissue, CSCs can give rise to progenitor cells that differentiate into various types of heterogeneous colorectal cancers. Moreover, CSCs are highly drug-resistant, making them one of the main causes of colorectal cancer malignancy and recurrence^3,4,7,8^.

Several molecular markers have been identified for the characterization of colorectal CSCs, including surface molecules such as CD133, CD44, CD24, LGR5, and EpCAM^2,9–15^ and drug efflux transporters like ALDH1 and ABCG2^16–19^. Among those markers, CD24, CD44, LGR5, and ALDH1 are target genes of Wnt/β-catenin signaling as well as key hallmarks of colorectal CSCs^2,11,12,18,20–22^. Emerging data suggest that the Wnt signaling is essential to colorectal CSC function and that β-catenin-mediated regulation of target genes is closely related to colorectal cancer malignancy.

The Wnt signaling cascade is composed of various proteins from Wnt, the ligand protein, to β-catenin, the key transcriptional coactivator^23^. Dysregulated Wnt signaling caused by mutations in Wnt signaling components is crucial for cancer initiation, late-stage cancer, and metastasis^24–27^. Because β-catenin is the most downstream effector protein in the Wnt signaling pathway, its final concentration in the nucleus and its proper recruitment to target-gene promoters are important issues in cancer progression. Recent studies have shown that not only genetic mutations but also epigenetic changes such as DNA methylation and histone modifications are associated with the Wnt signaling pathway^28,29^. In particular, the histone demethylase JMJD2C regulates sphere formation by modulating the recruitment of β-catenin to target genes in CRCs^30^.

JIB-04 is a small molecule that inhibits the demethylase activity of the Jumonji family of histone lysine demethylases (KDMs) by a novel mechanism. JIB-04 is a pan-selective inhibitor known to reduce cancer growth in lung cancer and prostate cancer cell lines^31^. Although recent studies have shown that JIB-04 induces cell death in drug-resistant brain cancer and lung cancer cells^32,33^, there is currently no experimental evidence that JIB-04 has similar effects on colorectal CSCs^34^.

In this present study, we aimed to identify the small molecule(s) that selectively target CSCs in colorectal cancer by screening inhibitors for various epigenetic pathways. From our primary drug screening, we identified JIB-04 as most effective drug in inhibition of tumorsphere formation. In light of several lines of evidence suggesting that JIB-04 is a potential anti-CSC drug, we examined the efficacy and mechanism of JIB-04 action on the clonal expansion, self-renewal, and differentiation of human colorectal CSCs. We found that JIB-04 treatment attenuated tumorsphere initiation and growth, CSC marker expression, and clonogenic proliferation in several colorectal cancer cell lines. At the molecular level, JIB-04 down-regulated the expression of Wnt/β-catenin-regulated target genes associated with colorectal CSC progression, possibly by interfering with the interaction between JMJD2 and β-catenin. Collectively, our results suggest that JIB-04 may be a novel therapeutic agent for colorectal cancer.

## Results

### Effect of JIB-04 on the viability and cell cycle of human colorectal cancer cells

**Figure 1:**
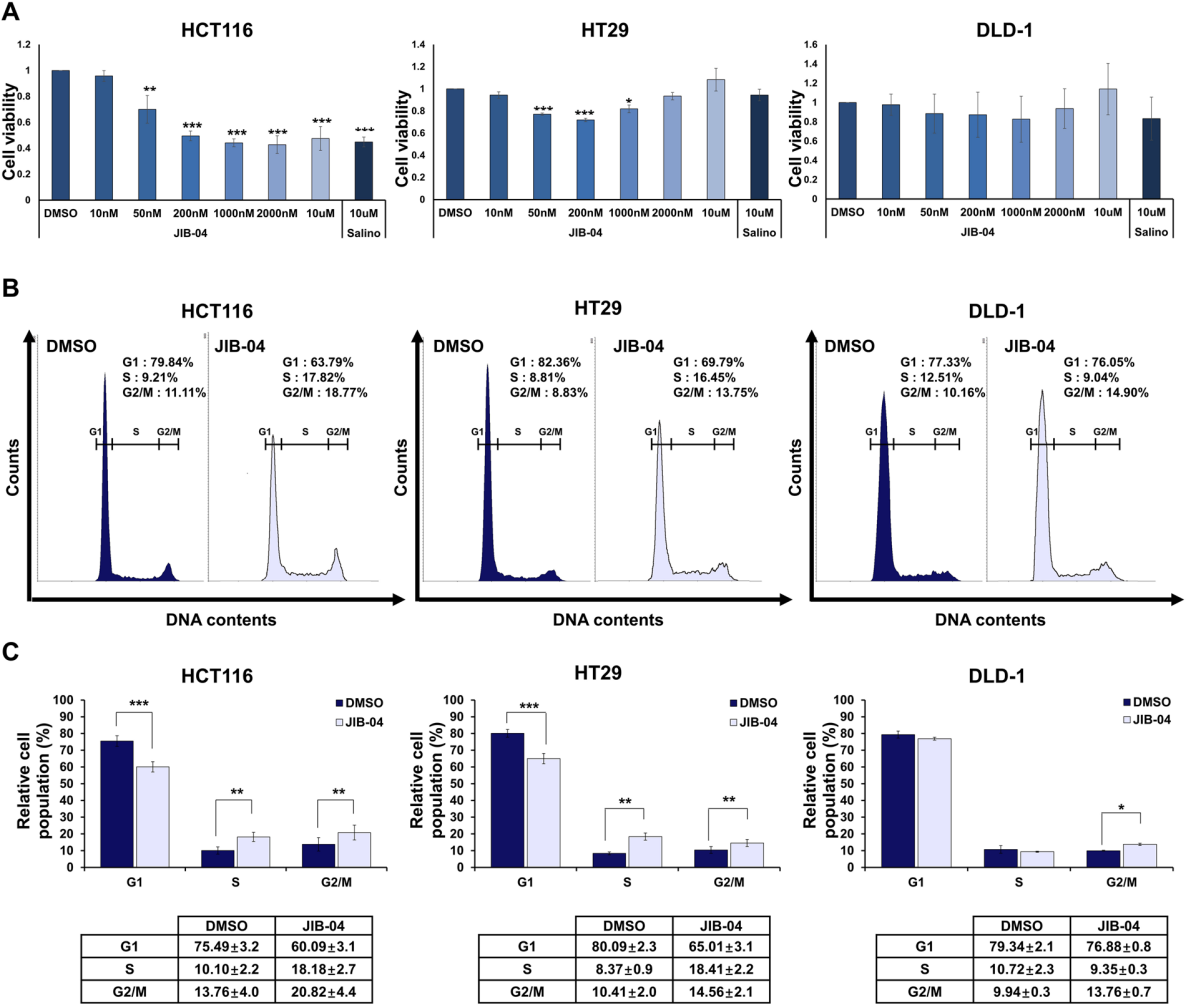
Effect of JIB-04 on cell viability and the cell cycle. (**A**) Viability of cells cultured after treatment with various doses of JIB-04 or 10 µM salinomycin for 24 hr. Cell viability of DMSO-treated cells was set as 1 (n = 3). (**B**) Representative histograms of the cell cycle distribution in three colorectal cancer cell lines after treatment with DMSO (control) or JIB-04 for 24 hr. Cell were stained with propidium iodide to detect their DNA content. (**D**) Bar graph representing relative cell populations in cell cycle phases G_1_, S, and G_2_/M. *p < 0.05, **p < 0.01, ***p < 0.001, compared with DMSO.

Because many lines of evidences support the concept that deregulation of various epigenetic pathways might contribute to cancer initiation and tumorigenesis^34^, we screened specific inhibitors of epigenetic modifiers for their effects on tumorsphere forming ability of CSCs in human colorectal cancer cells. Among them, JIB-04, a small molecule histone demethylase inhibitor, was most effective in inhibition of tumorsphere formation (Supplementary Fig. S1A). JIB-04 was previously reported to have selective anticancer activity in lung cancer^31^, but its effects on colorectal cancer cells have not been elucidated. So, we first determined the influence of JIB-04 on the viability and cell cycle progression of human colorectal cancer cells. When we treated HCT116 cells with JIB-04 for 24 h, the cell viability decreased as the concentration of JIB-04 increased, with a maximum reduction in viability of about 50% compared with the viability of the DMSO-treated control cells (Fig. 1A). In contrast, the JIB-04 treatment had a much smaller effect on the viability of HT29 cells and had no significant effect on the viability of DLD-1 cells. In all three cell lines, the maximum effect of JIB-04 on cell viability was similar to the effect of salinomycin, which was used as a control drug because of its known ability to target colorectal CSCs^35^. Although the short-term effects of JIB-04 on cell viability were not very strong, continuous treatment with JIB-04 over several days strongly blocked cell viability (Supplementary Fig. S2A). In addition, we investigated the effect of JIB-04 on cell cycle progression in colorectal cancer cells by FACS analysis. Compared with DMSO-treated controls, JIB-04-treated HCT116 and HT29 cells showed increased S-phase and G_2_/M-phase subpopulations and decreased G_1_-phase subpopulations (Fig. 1B,C). JIB-04-treated DLD-1 cells showed an increase in the G_2_/M subpopulation but no significant changes in the G_1_ and S subpopulations compared with DMSO-treated controls.

### JIB-04 abrogated the stemness of colorectal CSCs

**Figure 2:**
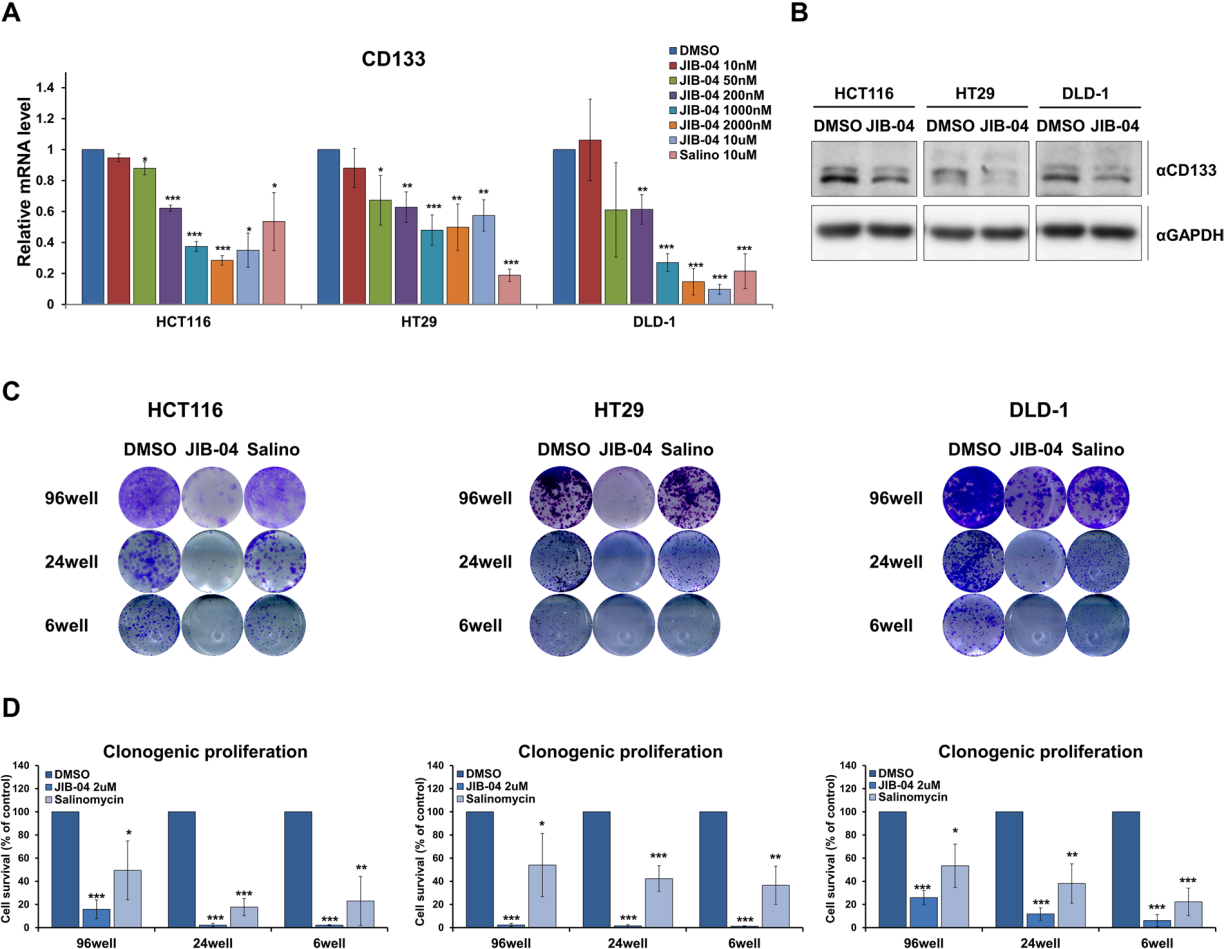
The self-renewal ability of colorectal CSCs was decreased by JIB-04. (**A**) mRNA expression level of CD133 in colorectal cancer cells cultured after treatment with escalating doses of JIB-04 or 10 µM of salinomycin for 24 hr. The mRNA level of CD133 was normalized to that of GAPDH. The expression level of the DMSO-treated cells was set as 1 (n = 3). (**B**) Protein expression of CD133 in colorectal cancer cells after treatment with 2 µM JIB-04 for 24 h was confirmed by western blot analysis. GAPDH was used as a loading control. (**C**) Clonogenic proliferation of colorectal cancer cells treated with DMSO (control), 2 µM JIB-04, or 10 µM Salinomycin. Cells were stained with crystal violet (n = 3). (**D**) Quantification of the colony-containing areas from panel C. *p < 0.05, **p < 0.01, ***p < 0.001, compared with DMSO.

To determine whether JIB-04 abrogates the stemness of colorectal CSCs, we examined the expression level of the colorectal CSC marker CD133 in HCT116, HT29, and DLD-1 cells after treatment with various concentrations of JIB-04 for 24 h. The CD133 mRNA expression level generally decreased in all three JIB-04-treated cell lines in a dose-dependent manner, regardless of the effects of the same JIB-04 treatments on cell viability, suggesting that the JIB-04 treatment resulted in a significant reduction of the colorectal CSC population (Fig. 2A). The JIB-04 treatment also reduced the CD133 protein expression level in all three cell lines (Fig. 2B).

Next, because the self-renewal ability of CSCs enables them to survive and form colonies without neighbor cells, we investigated clonogenic proliferation of JIB-04-treated cells to determine the effect of JIB-04 on the self-renewal ability of CSCs. After culturing the HCT116, HT29 and DLD-1 cells with DMSO, JIB-04, or salinomycin for 24 h, we re-seeded the cells in at a density of 10^3^ cells per well in tissue culture plates (6, 24, and 96 wells), grew the cells for 10 days, and then visualized them by crystal violet staining^36^. Although treatment with JIB-04 did not affect the viability of HT29 and DLD-1 cells, the 2 µM JIB-04 treatment dramatically abated the clonogenic proliferation of those cells after the cells were re-seeded. The 2 µM JIB-04 treatment caused greater inhibition of clonogenic proliferation than the treatment with 10 µM salinomycin, a well-studied CSC targeting agent. The areas of positively stained colonies on the plates decreased with decreasing cell density (Fig. 2C). Quantitative analysis showed that the JIB-04 treatment significantly reduced the survival of the cells (Fig. 2D). Those results indicate that JIB-04 selectively targeted CSCs and blocked the self-renewal ability of those cells.

### JIB-04 attenuated the tumor-initiating and growth/relapse abilities of tumorspheres

**Figure 3:**
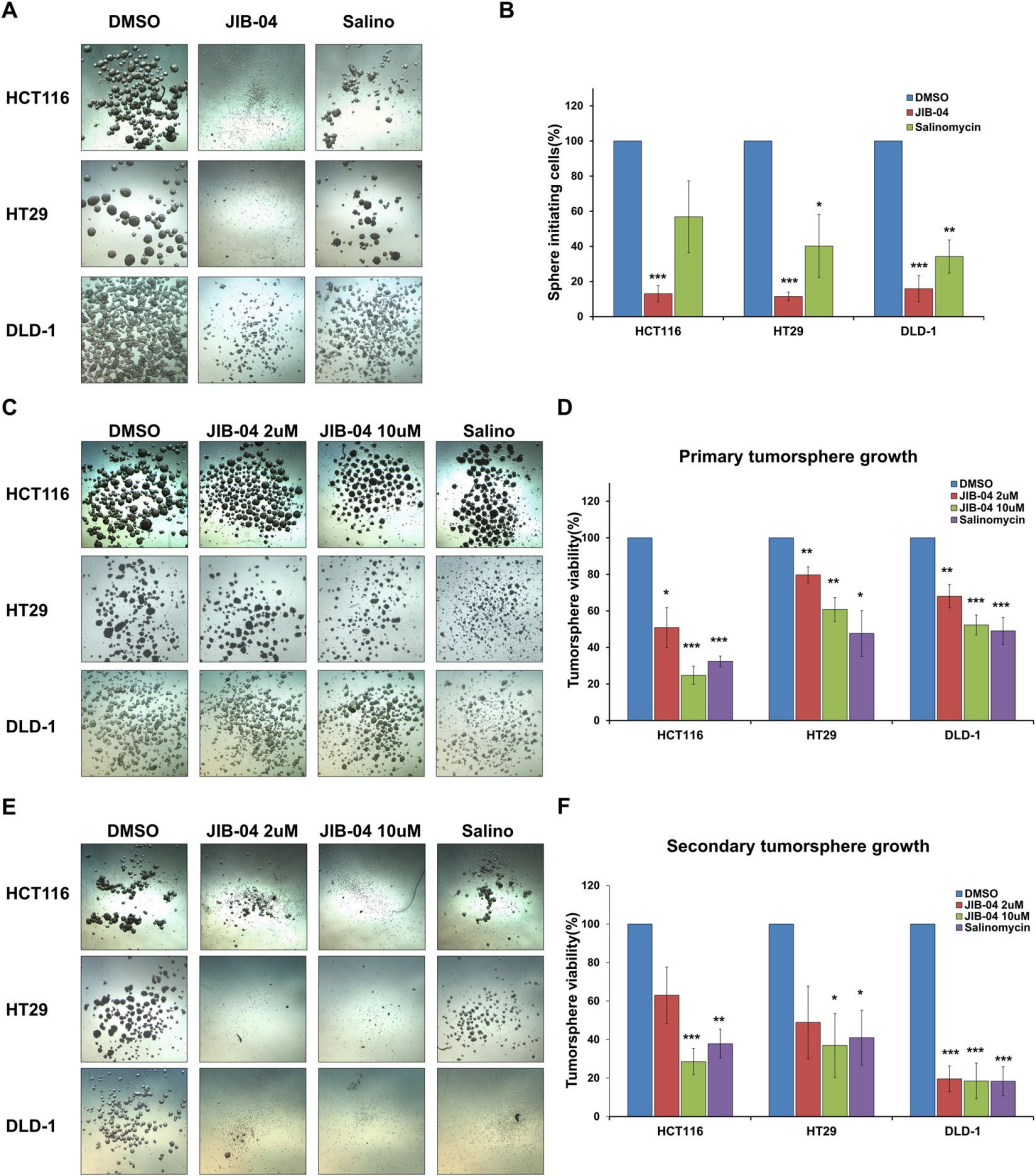
Effect of JIB-04 on the tumor-initiating ability and growth/relapse of tumorspheres. (**A**) Phase contrast image of tumorspheres derived from cells cultured with DMSO (control), 2 µM JIB-04, or 10 µM salinomycin for 24 h. (**B**) Percentages of sphere-initiating cells measured by CCK assay. The number of DMSO-treated cells was set as 100 (n = 3). (**C**) Phase contrast image of primary tumorspheres after treatment with DMSO (control), 2 µM JIB-04, 10 µM JIB-04, or 10 µM salinomycin for 2 days. (**D**) Viability of primary tumorspheres measured by CCK assay. The number of DMSO-treated cells was set as 100 (n = 3). (**E**) Phase contrast image of secondary tumorspheres developed from primary tumorspheres without drug treatment for 12 days. (**F**) Viability of secondary tumorspheres measured by CCK assay. *p < 0.05, **p < 0.01, ***p < 0.001, compared with DMSO.

CSCs have an ability to initiate and drive tumorigenesis, which contributes to the chance of relapse. We performed tumorsphere formation assays to explore the effect of JIB-04 on tumor initiation, growth, and relapse. To evaluate whether JIB-04 treatment weakens the tumor-initiating ability of CSCs, we cultured HCT116, HT29, and DLD-1 tumorspheres on adherent culture dishes after treatment with DMSO, 2 µM JIB-04, or 10 µM salinomycin for 24 h. After 7 days, the DMSO-treated control cells developed dense and round-shaped tumorspheres, but the JIB-04-treated cells failed to form tumorspheres, remaining almost as single cells (Fig. 3A). Quantitative analysis by CCK assay showed that the JIB-04 treatment reduced the percentages of sphere-initiating cells to about 10% of that of control cells and was more effective than the salinomycin treatment in inhibiting the initiation of tumorspheres (Fig. 3B). Furthermore, to determine the ability of JIB-04 to inhibit tumorsphere growth, we induced tumorsphere formation for 5 days and subsequently treated the spheres with DMSO (control), 2 µM JIB-04, 10 µM JIB-04, or 10 µM salinomycin for 2 days. The JIB-04 treatments reduced the size of the tumorspheres and caused the tumorspheres to dissociate (Fig. 3C). Consistent with those morphological changes, CCK assays confirmed that the JIB-04 treatments reduced the viability of the tumorsphere cells, suggesting that JIB-04 inhibited the growth of the tumorspheres by targeting CSCs (Fig. 3D). Then, to examine effect of JIB-04 on relapse, we developed secondary tumorspheres by culturing the surviving cells shown in Fig. 3C in standard stem cell medium without additional drug treatment for 12 days. The control cells derived from DMSO-treated primary tumorspheres were able to form secondary tumorspheres that were similar to the primary tumorspheres. On the contrary, the surviving cells derived from JIB-04-treated primary tumorspheres mostly developed secondary tumorspheres with irregular shapes (Fig. 3E). The numbers of viable cells in the secondary tumorspheres derived from JIB-04-treated primary tumorspheres were also decreased, implying that the regrowth of tumorspheres was efficiently suppressed by the pre-treatment with JIB-04.

### JIB-04 reduced the tumorigenic activity of colorectal CSCs ***in vivo***

**Figure 4:**
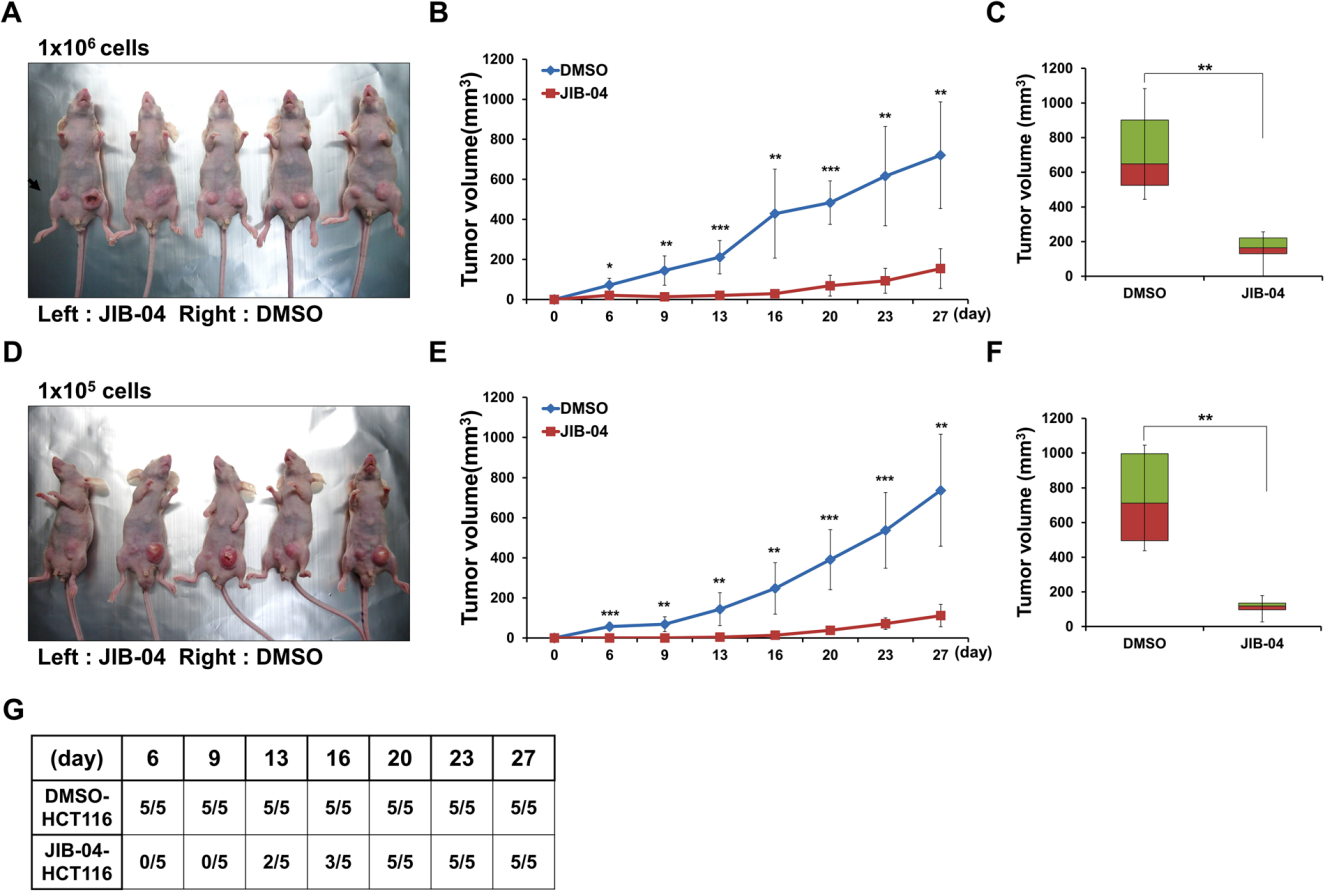
JIB-04 reduced the tumorigenic activity of colorectal CSCs *in vivo*. (**A**–**F**) BALB/c nude mice were injected subcutaneously with the indicated number of HCT116 cells (left; 2 µM JIB-04-treated cells, right; vehicle-treated cells) (**A** and **D**). Tumor size was measured on the indicated days after injection of HCT116 cells (**B** and **E**). The bar graph reflects the mean ± SD volume of developing tumors at 27 days after injection (**C** and **F**). (**G**) The numbers of mice developing tumors at the indicated days after inoculation of 1 × 10^5^ tumor cells. (n = 5) *p < 0.05, **p < 0.01, ***p < 0.001, compared with DMSO.

To assess the anti-tumorigenic activity of JIB-04 *in vivo*, we treated HCT116 cells with 2 µM JIB-04 or DMSO for 48 h and then injected 1 × 10^6^ or 1 × 10^5^ of the treated cells subcutaneously into nude mice. In tumors developed from JIB-04-treated cells, the rate of tumor growth (Fig. 4A–E) and the final tumor volume (Fig. 4C–F) were markedly reduced compared with those in tumors developed from DMSO-treated control cells. The injection of 1 × 10^5^ JIB-04-treated cells resulted in no tumor development in the early days of the experiment (Fig. 4G).

Because of their strong tumorigenic activities, even a small number of CSCs can generate a tumor. Therefore, we carried out xenograft experiments with by injecting 100 cells into NOD/SCID mice. As previously observed in the xenograft assays using nude mice (Fig. 4), the JIB-04 treatment significantly reduced the tumor growth rate and final volume in the NOD/SCID mice (Supplementary Fig. S3A and B).

### JIB-04 treatment inhibited colorectal cancer cell invasion and migration

**Figure 5:**
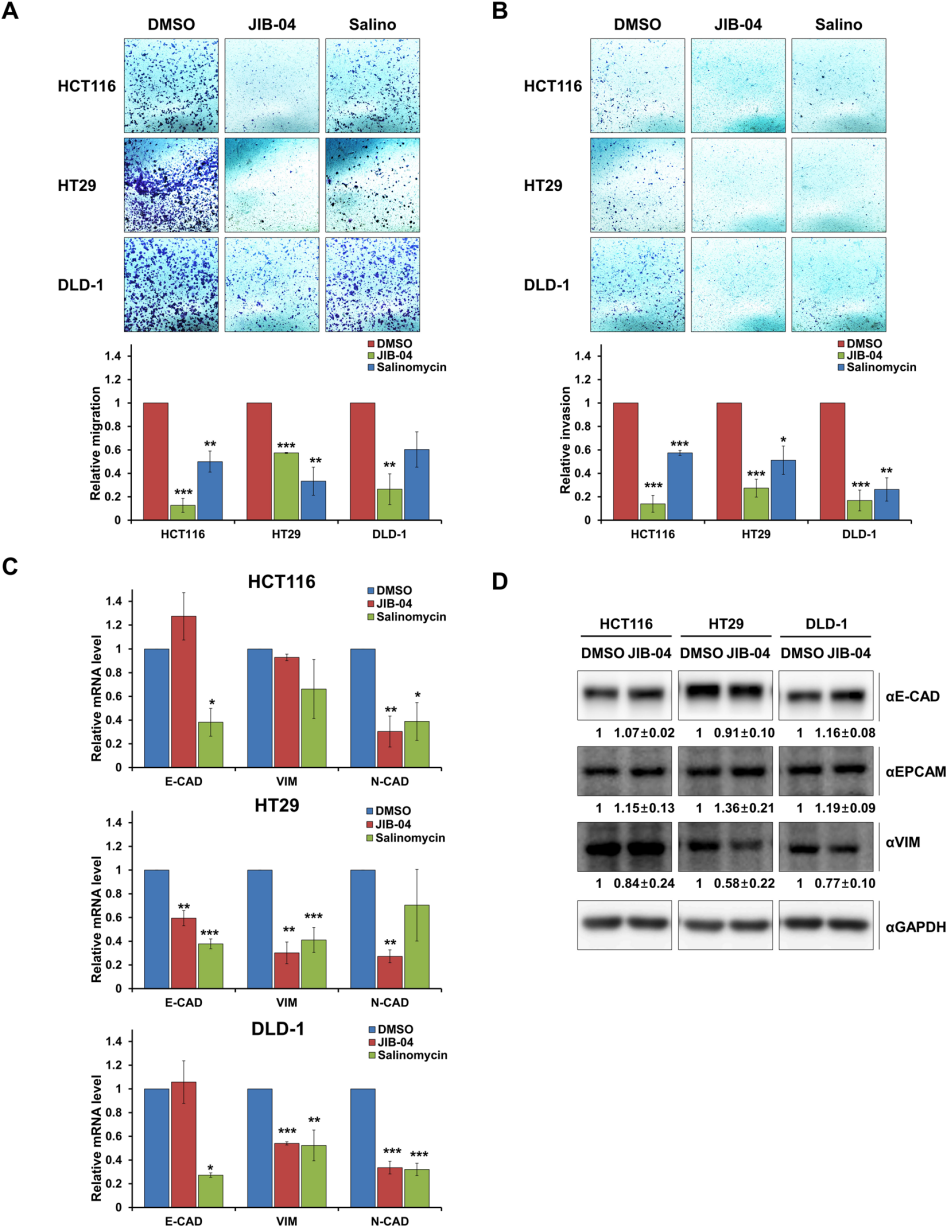
JIB-04 inhibited cell invasion and migration in colorectal CSCs. (**A**) Cell migration was investigated by transwell assay after treatment with DMSO, 2 µM JIB-04, or 10 µM salinomycin for 24 h (n = 3). (**B**) Invasive ability was examined in transwells coated with Matrigel after treatment with DMSO, 2 µM JIB-04, or 10 µM salinomycin for 24 h (n = 3). (**C**) mRNA expression of E-cadherin, vimentin, and N-cadherin after treatment with DMSO, 2 µM JIB-04, or 10 µM salinomycin for 24 h. The mRNA levels of the target genes were normalized to that of GAPDH. The expression level of DMSO-treated cells was set as 1 (n = 3). (**D**) Protein expression of E-cadherin, EpCAM, and vimentin after treatment with DMSO, 2 µM JIB-04, or 10 µM salinomycin for 24 h. GAPDH was used as a loading control. *p < 0.05, **p < 0.01, ***p < 0.001, compared with DMSO.

We performed transwell assays to determine if JIB-04 affects the migration and invasion abilities of colorectal cancer cells. HCT116, HT29, and DLD-1 cells treated with JIB-04 displayed reduced migration and invasion compared with control cells treated with DMSO or salinomycin (Fig. 5A and B). Analysis of the expression of epithelial-to-mesenchymal transition (EMT) markers revealed that the mRNA expression level of E-cadherin was increased in HCT116 and DLD-1 while those of vimentin and N-cadherin were decreased in JIB-04-treated cells compared with those in DMSO-treated control cells (Fig. 5C). In terms of protein expression, the JIB-04 treatment resulted in the up-regulation of epithelial markers (E-cadherin and EpCAM) and the down-regulation of mesenchymal marker (vimentin; Fig. 5D). Those results suggest that JIB-04 impedes cell migration and invasion by dysregulating EMT-related gene expression.

### Transcriptome analysis by RNA sequencing after treatment with JIB-04

**Figure 6:**
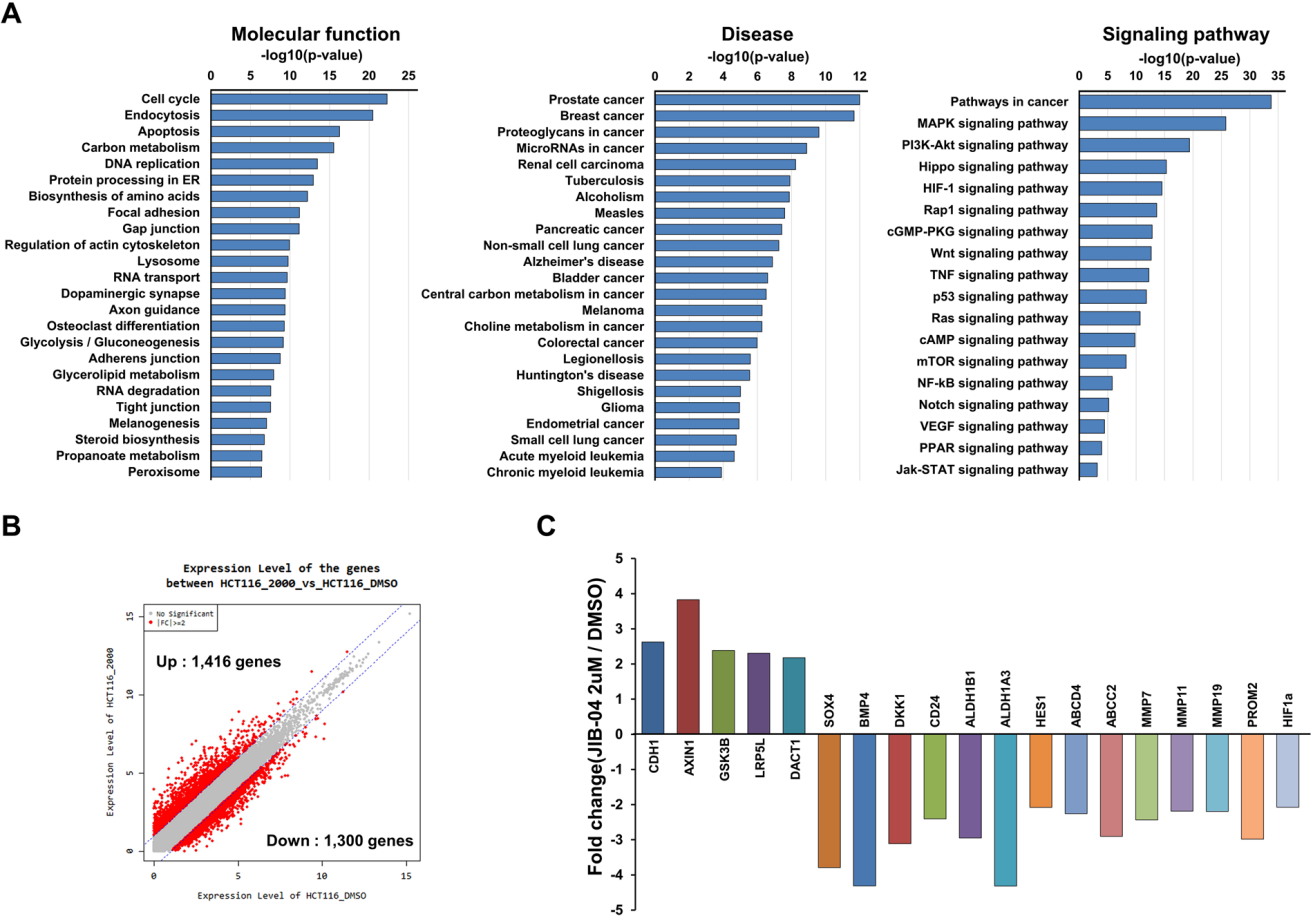
Transcriptome analysis of HCT116 cells after treatment with JIB-04. (**A**) KEGG pathway enrichment analysis. Raw p-values were calculated by modified Fisher’s exact test. The pathway enrichment scores (−Log10 P-value) among the differentially expressed genes are shown. (**B**) Scatter plot of differential gene expression between 2 µM JIB-04 treatment and DMSO treatment. (**C**) Differential expression of genes related to the Wnt/β-catenin signaling pathway between 2 µM JIB-04 treatment and DMSO treatment.

To understand the mechanism by which JIB-04 affects colorectal CSCs, we carried out RNA sequencing analysis using RNA extracts from HCT116 cells after treatment with 2 µM JIB-04 for 24 h. KEGG pathway analysis revealed that the JIB-04 treatment altered the expression of genes that are involved in the cell cycle, apoptosis, and DNA replication and are also related to several cancers including colorectal cancer. JIB-04 also altered the expression of genes involved in various signaling pathways such as the MAPK signaling pathway, the PI3K-Akt signaling pathway, and the Wnt signaling pathway (Fig. 6A). JIB-04 treatment resulted in the up-regulation of 1,416 genes and the down-regulation of 1,300 genes (Fig. 6B). Among those genes, we focused on genes related to the Wnt/β-catenin signaling pathway, because the abnormal regulation of Wnt/β-catenin signaling is known as a major cause of colorectal cancer^37^. The JIB-04 treatment increased the expression of genes involved in the inhibition of β-catenin signaling, including AXIN1, GSK3β, LRP5L, and DACT1, whereas it decreased the expression of β-catenin target genes, including BMP4, DKK1, CD24, ALDH1B1, ALDH1A3, and SOX4. In addition, JIB-04 caused a decrease in the expression of CSC markers (HES1, ABCD4, ABCC2, MMP7, MMP11, MMP19, and PROM2; Fig. 6C).

### JIB-04 inhibited Wnt/β-catenin signaling in colorectal cancer cells

**Figure 7:**
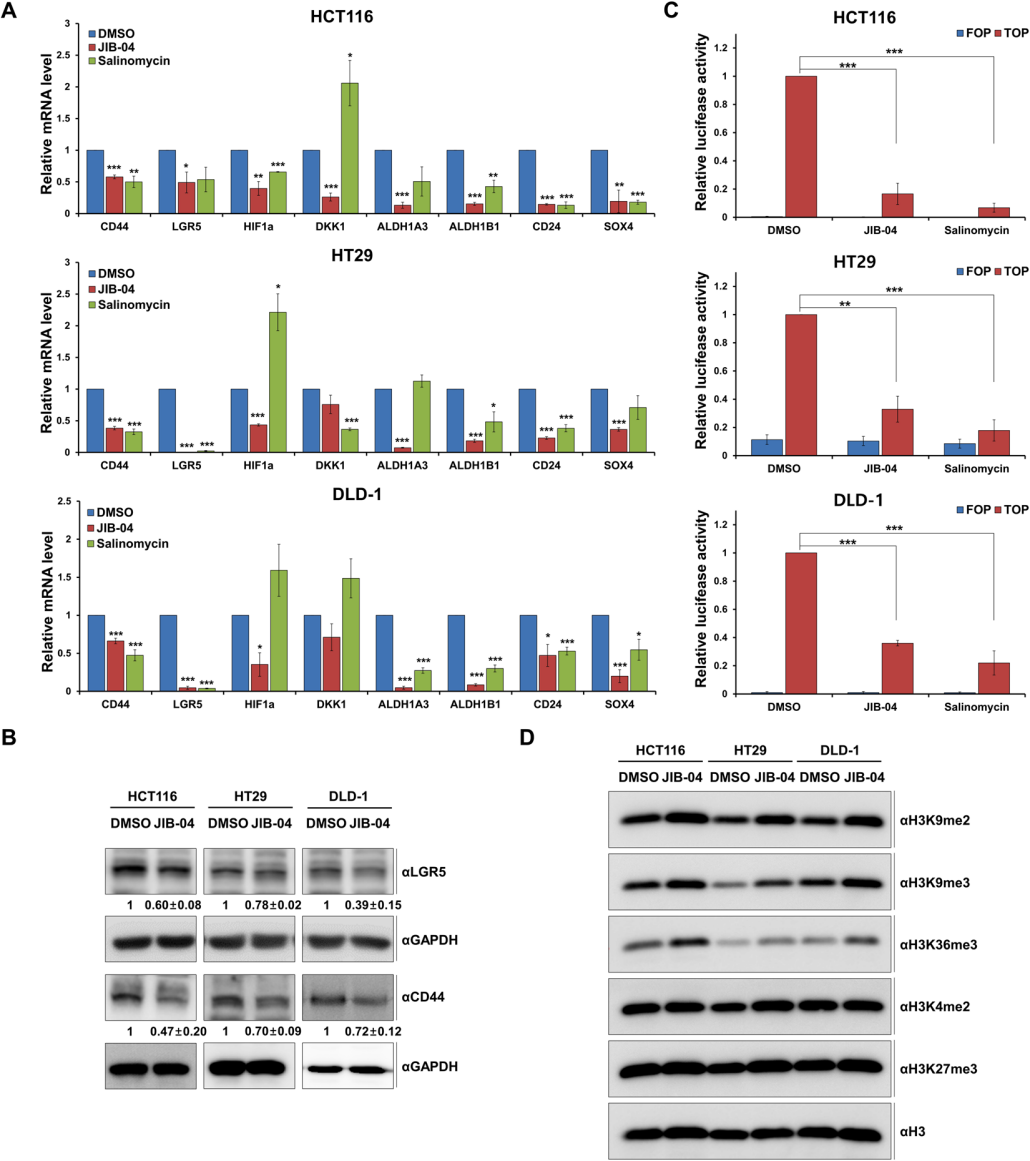
JIB-04 inhibited the β-catenin signaling pathway in colorectal cancer cells. (**A**) mRNA expression levels of β-catenin target genes associated with CSCs after treatment with DMSO, 2 µM JIB-04, or 10 µM salinomycin for 24 h. The mRNA levels of the target genes were normalized to that of GAPDH. The expression level of DMSO-treated cells was set as 1 (n = 3). (**B**) Protein expression of LGR5 and CD44 after treatment with DMSO or 2 µM JIB-04 for 24 h. GAPDH was used as a loading control. (**C**) TOP flash reporter assay of β-catenin-based promoter activity after treatment with DMSO, 2 µM JIB-04, or 10 µM salinomycin for 24 h. Cells were transfected with TOP or FOP flash plasmids and renilla luciferase plasmid. Luciferase activity was measured 48 h after transfection and normalized to that of renilla. The luciferase activity of DMSO-treated cells was set as 1 (n = 3). (**D**) Western blot analysis of histone methylation levels after treatment with DMSO or 2 µM JIB-04 for 24 h. H3 was used as a loading control. *p < 0.05, **p < 0.01, ***p < 0.001, compared with DMSO.

To validate the effects of JIB-04 on the mRNA expression of β-catenin target genes, we measured the mRNA levels of CSC-associated genes (CD44, LGR5, DKK1, ALDH1A3, ALDH1B1, CD24, and SOX4) by qRT-PCR in three different colorectal cancer cell lines after treament with JIB-04. The JIB-04 treatment reduced the mRNA expression of those genes in all three cell lines. In addition, the JIB-04 treatment significantly reduced the expression of HIF1a, which is known to be related to the β-catenin pathway (Fig. 7A). Next, we confirmed the protein expression of LGR5 and CD44. Consistent with the mRNA expression, the protein expression of LGR5 and CD44 was reduced by JIB-04 (Fig. 7B). Based on those results, we hypothesized that JIB-04 down-regulated the expression of β-catenin target genes via the negative control of β-catenin. The JIB-04 treatement did not change the level of β-catenin protein expression (Supplementary Fig. S4A), however, suggesting that our results might be not caused by reduced β-catenin expression, although long-term JIB-04 treatment did decrease the β-catenin protein level (Supplementary Fig. S4B).

To further investigate the potential regulatory effects of JIB-04 on the transcriptional activity of β-catenin on TCF/LEF, we performed a TOP/FOP flash assay. Our results showed that JIB-04 treatment inhibited the TOP signals (Fig. 7C), supporting the hypothesis that the JIB-04-mediated effects on the expression of β-catenin target genes were generated via the down-regulation of the β-catenin pathway. Because JIB-04 is an inhibitor of Jumonji domain-containing histone demethylases, we investigated global histone H3 methylation levels to clarify which Jumonji demethylases in colorectal cancer cells were influenced by JIB-04. We found that JIB-04 up-regulated the di-methylation and tri-methylation of H3K9 as well as the tri-methylation of H3K36 in HCT116, HT29, and DLD-1 cells, suggesting that JIB-04 inhibits the JMJD2 histone demethylase family in colorectal cancer cells (Fig. 7D). It was previously reported that JMJD2B and JMJD2C interact with β-catenin to recruit β-catenin to the promoters of target genes involved in the EMT and Wnt pathways^30,38,39^. Taken together, our data suggest that JIB-04 disrupts Wnt/β-catenin signaling in colorectal CSCs by inhibitinig the JMJD2 histone demethylase family.

## Discussion

The small molecule JIB-04 was recently reported as a novel, pan-selective inhibitor of Jumonji demethylases that has specific anticancer activities in human lung and prostate cancer cells^31^. More recently, JIB-04 was also shown to induce apoptosis and inhibit growth in drug-resistant lung cancer and glioblastoma cells^32,33^. Although drug resistance is one of the most important features of CSCs, the role of JIB-04 in CSC function has not been clarified. In addition, there are no reports of the role of JIB-04 in colorectal cancer cells. In this study, we identified a novel role of JIB-04 in the survival and maintenance of colorectal CSCs using three different colorectal cancer cell lines.

Although JIB-04 treatment did not markedly reduce the overall viability of the colorectal cancer cells (Fig. 1A), it significantly decreased a representative CSC marker, CD133, in a dose-dependent manner (Fig. 2A,B). In addition, we observed that JIB-04 treatment diminished the self-renewal, tumor-initiating ability, and tumorsphere growth of the colorectal cancer cells (Fig. 2C,D; Fig. 3A–D). Because those features are crucial to the function of colorectal CSCs, our results suggest that JIB-04 might be used to specifically target colorectal CSCs. JIB-04 treatment interfered with the formation of secondary tumorspheres as well as the growth of primary tumorspheres (Fig. 3C–F), suggesting that JIB-04 not only reduced the number of colorectal CSCs but also changed the characteristics of the colorectal CSCs. The JIB-04 treatment also induced G_2_/M arrest in colorectal cancer cells (Fig. 1B,C) and effectively reduced the size of xenograft tumors in both of BALB/c nude mice and NOD/SCID mice (Fig. 4). Collectively, our results show that JIB-04 has potent and selective anti-proliferative effects on colorectal cancer cells and effectively eliminates some of the stemness of colorectal CSCs.

We showed that JIB-04 treatment affects numerous molecular functions, disease mechanisms, and pathways (Fig. 6). JIB-04 altered the expression levels of genes related to colorectal cancer as well as various other cancers, suggesting that JIB-04 may have anti-CSC activity in cancers other than colorectal cancer. JIB-04 affected several signaling pathways, but because hyperactivation of Wnt/β-catenin signaling is a hallmark of both the early stages and the late stages of colorectal cancer development^24–27^, we focused on the Wnt signaling pathway. Recent studies showed that Wnt/β-catenin signaling acts as a key regulator of colorectal CSCs by regulating EMT and CSC marker genes^23,25,37,40,41^. Our results showed that JIB-04 significantly reduced the expression of Wnt target genes and CSC markers including CD44, LGR5, ALDH, HIF1a, and SOX4 (Figs 6C and 7A,B). JIB-04 inhibited the transcription of β-catenin target genes as confirmed by TOP/FOP flash luciferase assays (Fig. 7C).

Although the mechanism underlying the JIB-04-mediated inhibition of β-catenin-dependent transcription will require further investigation to uncover, we can suggest one possible mechanism. Three different histone modifications (H3K9me2, H3K9me3, and H3K36me3) were largely increased in response to JIB-04 treatment in all three colorectal cancer cell lines (Fig. 7D). Those histone modifications are known to be main targets of the JMJD2 family proteins^42^. Therefore, we hypothesize that JIB-04 inhibits the JMJD2 family, which leads to the suppression of colorectal CSC activities. Consistent with that hypothesis, there are several reports that support the existence of a relationship between JMJD2 and β-catenin recruitment in cancers^30,38,39^. JMJD2B inhibition attenuated β-catenin recruitment to the gene promoter of vimentin, an EMT marker, inducing a decrease in gastric cancer metastasis^39^. Similarly, JMJD2C knockdown impaired sphere-forming ability through decreased β-catenin recruitment to the JAG1 gene promoter in colorectal cancer cells^30^. The effects of JIB-04 on the results of β-catenin promoter-based luciferase reporter assays in our study were almost same in all three cell lines despite the different mutation statuses of the three cells lines^20,43^. Therefore, JIB-04 likely acts downstream of the Wnt/β-catenin signaling pathway. Taken together, our results support the hypothesis that JIB-04 diminishes the stemness of colorectal CSCs by inhibiting JMJD2 histone demethylases, which are required for proper β-catenin recruitment to target genes.

JIB-04 strongly reduced the expression of LGR5 (leucine-rich repeat-containing G-protein-coupled receptor 5) in all three colorectal cancer cell lines (Fig. 7A,B). Although there are various CSC marker genes, several recent studies showed that LGR5 has significant effects on tumor growth and metastasis, especially in colorectal cancer^15,44–46^. One study found that the selective ablation of LGR5^+^ CSCs in organoids led to tumor regression, while the proliferation of LGR5^+^ CSCs may contribute to tumor regrowth^45^. In addition, LGR5^+^ CSCs were found to be critical for the formation and maintenance of liver metastasis derived from colorectal cancer^46^. Consistent with those observations, our results showed that JIB-04 treatment resulted in the dysregulation of EMT (Fig. 5) as well as decreased LGR5 expression. Thus, JIB-04 treatment might overcome the tumorigenic and metastasis-promoting actions of CRCs by dysregulating EMT ability and reducing LGR5 expression in CRCs.

Although our results demonstrated the main effect of JIB-04 on CSCs, long-term treatment with JIB-04 had dramatic inhibitory effects on the proliferation of colorectal cancer cells (Supplementary Fig. S2). Those inhibitory effects may be caused by a reduction in β-catenin protein levels via an unknown mechanism (Supplementary Fig. S4). JIB-04 does not modulate the proliferation and viability of normal cells (32), so JIB-04 might be an effective therapeutic agent for targeting total colorectal cancer cells, including non-CSC populations.

Only a few compounds have been reported to have specific inhibitory activity against colorectal CSCs. Salinomycin inhibits metastatic colorectal CSCs by interfering with Wnt/β-catenin signaling^35^. Very recently, the TNIK inhibitor NCB-0846 was shown to abrogate the stemness of colorectal CRCs by blocking Wnt signaling^47^. Our results clearly showed that the anti-CSC activity of JIB-04 is comparable to or greater than that of salinomycin. Overall, our results suggest that JIB-04 may be an excellent drug candidate against colorectal cancer.

## Materials and Methods

### Cell culture and drug treatment

We cultured HCT116, HT29, and DLD-1 cells under standard culture conditions in RPMI-1640 media (Welgene) containing 10% FBS (Atlas Biologicals) and 1 × penicillin-streptomycin (Corning). For the drug treatments, we dissolved JIB-04 (Cayman) and salinomycin (Cayman) in DMSO and diluted the solutions to the appropriate concentrations for the experiments. The following chemicals were used for the primary screening of anti-CSC agents: IOX1 (Cayman), SAHA (Cayman), trichostatin (TSA) (Cayman), EPZ5676 (APEXBIO), eosin Y disodium trihydrate(AMI-5) (Santa Cruz), 2-PCPA(Cayman), sirtinol (Cayman), pargyline (Sigma-Aldrich), paclitaxel (Cayman) 5-FU (Cayman), etoposide (Cayman), nocodazole (Sigma-Aldrich), calmidazolium chloride (Tocris Bioscience), beta-lapachone (Cayman).

### Tumorsphere formation

To investigate the tumor initiation ability of colorectal CSCs, we treated HCT116, HT29 and DLD-1 with DMSO, 2 µM JIB-04, or 10 µM salinomycin for 24 h and then seeded 2 × 10^4^ of the treated cells in stem cell medium (DMEM:F12, 20 ng/ml EGF, 20 ng/ml FGF, 5 µg/ml insulin, and 1× B27 supplement) on ultra-low-attachment six-well plates (Corning). After 7 days, we detected sphere-initiating cells using the Cell Counting Kit (CCK)-8 from Dojindo according to the manufacturer’s protocol.

To examine effect of JIB-04 on tumor growth, we cultured primary tumorspheres for 5 days and then treated them with DMSO, 2 µM JIB-04, 10 µM JIB-04, or 10 µM salinomycin for 2 days. We then measured the viability of the tumorspheres by CCK assay. To examine effect of JIB-04 on tumor recurrence, we trypsinized primary tumorspheres and re-seeded them without drug treatment to produce secondary tumorspheres. After 12 days, we measured the viability of the secondary tumorspheres by CCK assay.

### RNA extraction and real-time PCR

We extracted total RNA from HCT116, HT29 and DLD-1 using TRI-reagent LS (MRC) as previously reported^48^. We synthesized cDNA using a cDNA synthesis kit (Thermo Fisher Scientific) with oligo dT primers. We analyzed mRNA expression levels by quantitative real-time PCR (qRT-PCR) with SYBR Premix Ex Taq II (Takara, Japan) and an ABI7000 sequence detector (Applied Biosystems, USA) according to the manufacturers’ protocols using primer sets for the target genes described in Supplementary Table S1.

### Histone preparation and antibodies

We prepared histones as described previously^49^. Briefly, we lysed harvested HCT116, HT29 and DLD-1 cells after treatment of DMSO, 2 µM JIB-04, or 10 µM salinomycin for 24 h by mechanical shearing on a rotator in hypotonic lysis buffer at 4 °C. We extracted histones from the cell lysates with H_2_SO_4_ and precipitated them in TCA. After washing the precipitated histones with acetone, we dissolved the yields in distilled water.

We used the following antibodies for western blotting: anti-CD133 (Novus Biologicals, #NB120-16518), anti-E-cadherin (Cell signaling, #3195), anti-EpCAM (Cell signaling, #2929), anti-Vimentin (Cell signaling, #5741), anti-LGR5 (Novus Biologicals, #NBP1-28904), anti-CD44 (Santa Cruz Biotechnology, #sc-7297), anti-GAPDH (AbClon, #AbC-2003), anti-H3 (Abcam, #ab1791), anti-H3K9me2 (Abcam, #ab1220), anti-H3K9me3 (Millipore, #CS207324), anti-H3K36me3 (Abcam, #Ab9050), anti-H3K4me2 (Milipore, #07-030), and anti-H3K27me3 (Abcam, #Ab6002).

### TOP/FOP flash reporter assay

We transfected HCT116, HT29 and DLD-1 cells with TOP or FOP flash plasmids along with SV40-Renilla plasmid (control for transfection) using Lipofectamine 3000 (Invitrogen). On the following day, we transferred the cells to fresh media and treated them with DMSO, 2 µM JIB-04, or 10 µM salinomycin. Forty-eight hours after transfection, we detected luciferase activity using the Promega GLOMAX 20/20 system and dual-luciferase reporter assay system (Promega) according to the manufacturer’s protocol.

### Cell invasion assay and migration assay

We used 24-well culture plate with an 8.0-um transparent PET membrane separating the upper and lower halves of each well (Corning) to measure the migration ability and invasiveness of cells. After treating HCT116, HT29 and DLD-1 with DMSO, 2 µM JIB-04, or 10 µM salinomycin for 24 h for 24 h, we suspended 1 × 10^5^ treated cells in serum-free medium and added them to the upper chamber of the well containing the PET membrane. We filled the lower chamber with standard culture media containing 10% FBS as a chemoattractant. For the invasion assay, we covered the upper side of the membrane with Matrigel (BD). We then incubated the cultures for 24 h and stained the cells that passed through the membrane with crystal violet (Sigma Aldrich).

### Clonogenic proliferation

To investigate clonogenic proliferation, we seeded 1 × 10^6^ of HCT116, HT29 and DLD-1 cells in a 60 mm culture dish and treated them with DMSO, 2 µM JIB-04, or 10 µM salinomycin for 24 h. We then re-seeded 1 × 10^6^ treated cells per well in 96-well, 24-well, and 6-well plates (SPL) under standard culture conditions, grew them for 10 days, and then stained them with crystal violet. After scanning the culture plates, we quantified the results using ImageJ software.

### Cell cycle analysis

We treated HCT116, HT29 and DLD-1 with 2 µM JIB-04 for 24 h, fixed them in 70% ethanol for at least 30 min at 4 °C, and then stained them with 10 μg/ml propidium iodide solution containing 5ug/ml RNase A for 30 min. We analyzed the cell cycle profile by flow cytometry with fluorescence-activated cell sorting (FACS).

### RNA sequencing and pathway analysis

The RNA sequencing was processed by Macrogen, Inc. (Seoul, South Korea). RNA extracts from cells treated with DMSO or 2 µM JIB-04 for 24 h were subjected to cDNA library construction (TruSeq RNA Sample Prep Kit v2). The sample was checked for quality using FastQC v0.10.0 and then subjected to Illumina sequencing using the HiSeq. 4000 system. We aligned the sequencing reads to the reference genome using TopHat version 2.0.13 and bowtie2 2.2.3. We used Cufflinks version 2.2.1 to detect differences in expression between samples. We used the Kyoto Encyclopedia of Genes and Genomes (KEGG) database to determine the pathways of the differentially expressed genes. We selected significant pathways using Fisher’s exact test with the threshold of significance set by P-value and False Discovery Rate (FDR). The data discussed in this publication have been deposited in NCBI’s Gene Expression Omnibus and are accessible through GEO Series accession number GSE106407 (https://www.ncbi.nlm.nih.gov/geo/query/acc.cgi?acc=GSE106407).

### Animals

We obtained male 6-week-old BALB/c nude mice and NOD/SCID gamma (NSG) mice from Orient Bio (Seongnam, Korea) and The Jackson Laboratory (Bar Harbor, ME), respectively. All mice were maintained under SPF conditions in the AAALAC-accredited animal facility at the National Cancer Center.

### Subcutaneous colorectal cancer xenograft-tumor model

We treated HCT116 cells with vehicle (DMSO) or 2 µM JIB-04 for 2 days. After washing the cells, we suspended 1 × 10^5^ or 1 × 10^6^ cells in 100 μl PBS containing 50% Matrigel (BD Biosciences) and injected them subcutaneously into the left (JIB-04-treated cells) and right (DMSO-treated cells) flanks of BALB/c nude mice (6-week-old male). For the HCT-116-bearing NSG model, we treated HCT116 cells with vehicle (DMSO) or 2 µM JIB-04 for 1 day. After washing the cells, we suspended 100 viable cells in 100 μl PBS containing 50% Matrigel (BD Biosciences) and then inoculated the suspension into the subcutaneous tissues of male NSG mice.

We monitored the tumor size using a digital caliper and calculated the tumor volume according to the following formula: 0.5 × length (mm) × width (mm) (five mice per group). We performed all animal procedures according to the ARRIVE guidelines and the National Cancer Center guidelines for the care and use of laboratory animals. The protocol was approved by the Committee on the Ethics of Animal Experiments of the National Cancer Center (Permit Number: NCC-08-031).

## Electronic supplementary material

Supplementary data
