# Supplementary material for: JIB-04, A Small Molecule Histone Demethylase Inhibitor, Selectively Targets Colorectal Cancer Stem Cells by Inhibiting the Wnt/β-Catenin Signaling Pathway
Source: Sci Rep. 2018 Apr 26;8:6611. doi: 10.1038/s41598-018-24903-0 (PMC5919936; doi:10.1038/s41598-018-24903-0)
Supplement: Supplementary file 1 — Supplementary data [file 41598_2018_24903_MOESM1_ESM.pdf]

Supplementary Data

JIB-04, A Small Molecule Histone Demethylase Inhibitor, Selectively Targets Colorectal Cancer Stem Cells by Inhibiting the Wnt/β-Catenin Signaling Pathway

Min Seong Kim[1,2,*], Hye In Cho[1,2,*], Hee Jung Yoon[3], Ye-Hyeon Ahn[3], Eun Jung Park[3], Yan Hua Jin[4,5], and Yeun Kyu Jang[1,2]

Inventory of Supplemental Information

The Supplemental Information contains 6 additional figures, and 1 tables

Figure S1: Related to Figure 3

Figure S2: Related to Figure 1.

Figure S3: Related to Figure 4.

Figure S4: Related to Figure 7.

Figure S5: Uncropped gel image from Western blot: Fig 2B, Fig 5D and Fig 7B.

Figure S6: Uncropped gel image from Western blot: Fig 7D, Fig S4A and Fig S4B.

Table S1: Primer sequences for qRT-PCR

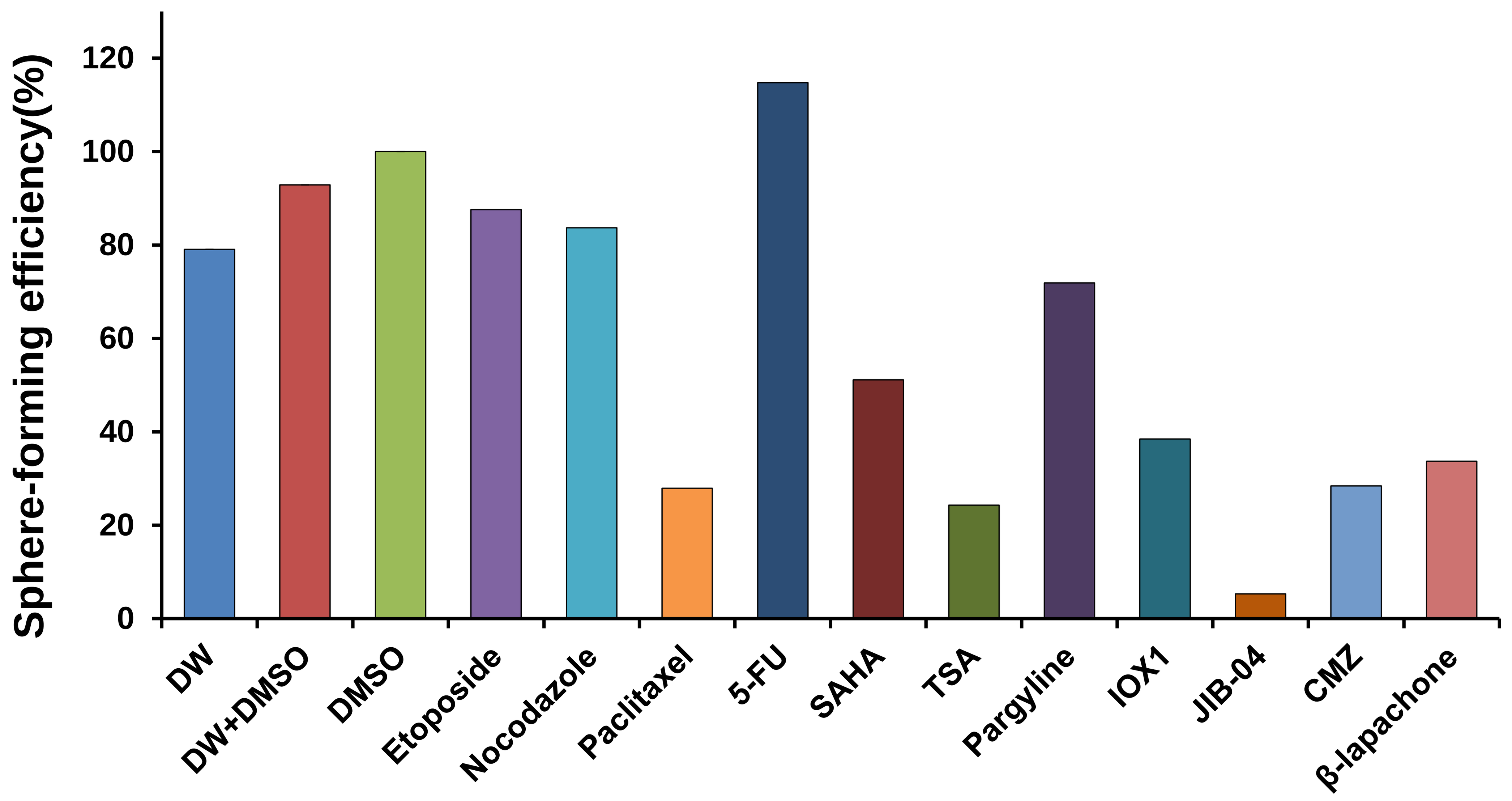

Supplementary Figure S1. Sphere-forming efficiency of HCT116 after treatment with 10μM of indicated drugs was analyzed by cell counting. Sphere-forming efficiency of DMSO-treated cells was set as 100. Symbols used: DW, distilled water; TSA, trichostatin A; CMZ, calmidazolium chloride.

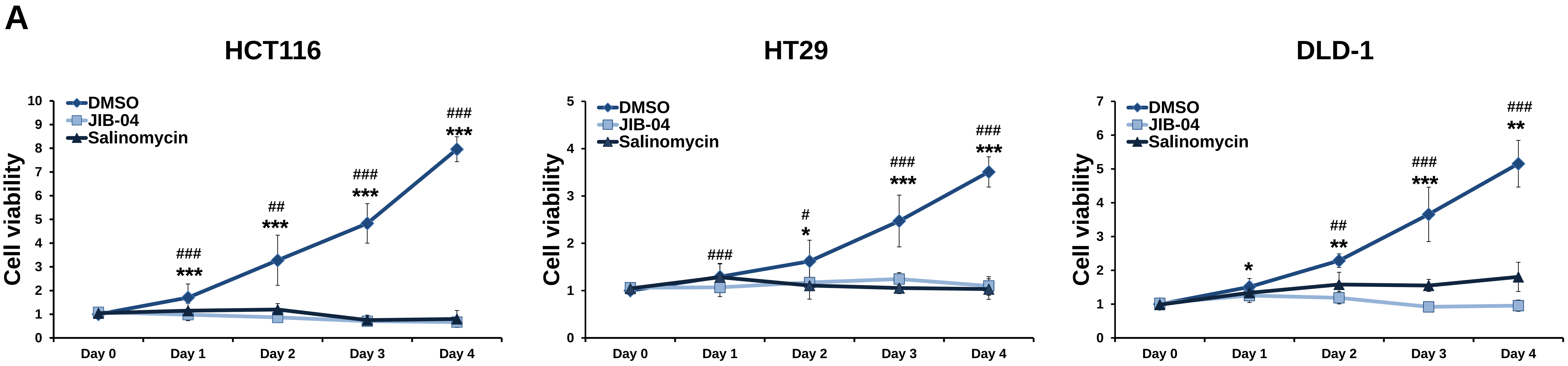

Supplementary Figure S2. Time course of the viability of cells cultured with DMSO, 2μM JIB-04, or 10μM salinomycin. Cell viability of DMSO-treated cells was set as 1 (n=3). *p < 0.05, **p < 0.01, ***p < 0.001 (DMSO vs. JIB-04). #p < 0.05, ##p < 0.01, ###p < 0.001 (DMSO vs. salinomycin).

A

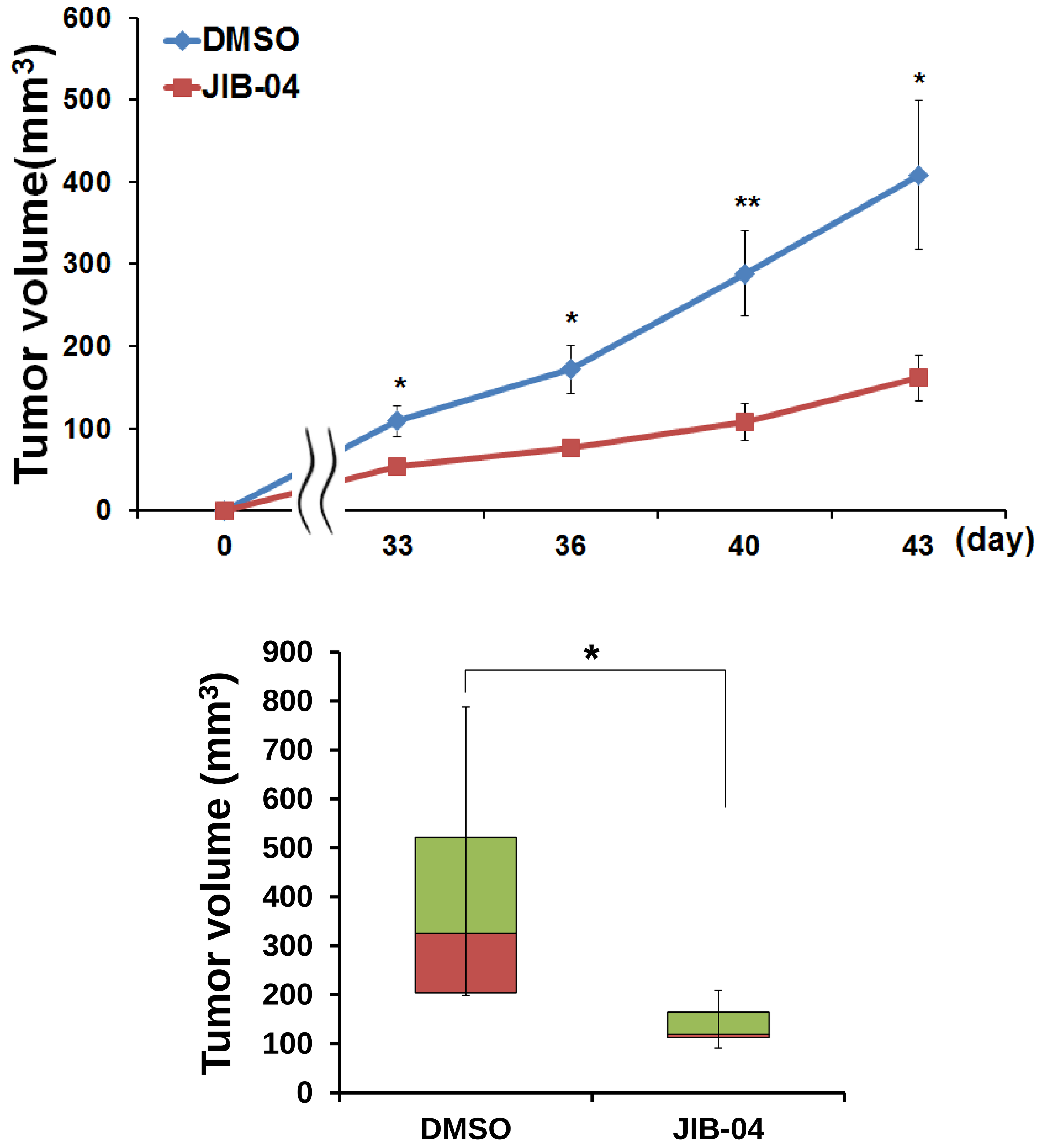

B

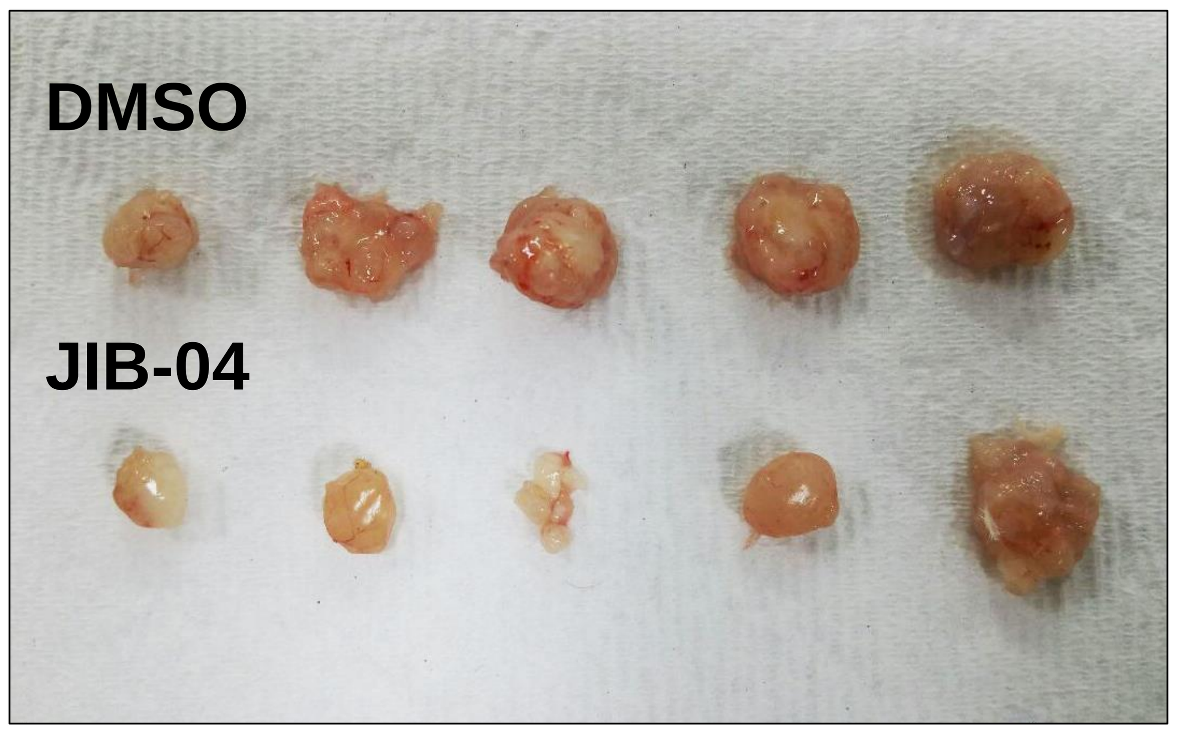

Supplementary Figure S3. A) HCT116 cells were treated with DMSO or 2 µM JIB-04 for 1 day and then injected into 6-week-old male NSG mice. Tumor volume was measured at the indicated days after the injection of HCT116 cells. B) Representative tumors were obtained 43 days after the injection of tumor cells. *p < 0.05, **p < 0.01, ***p < 0.001, compared with DMSO.

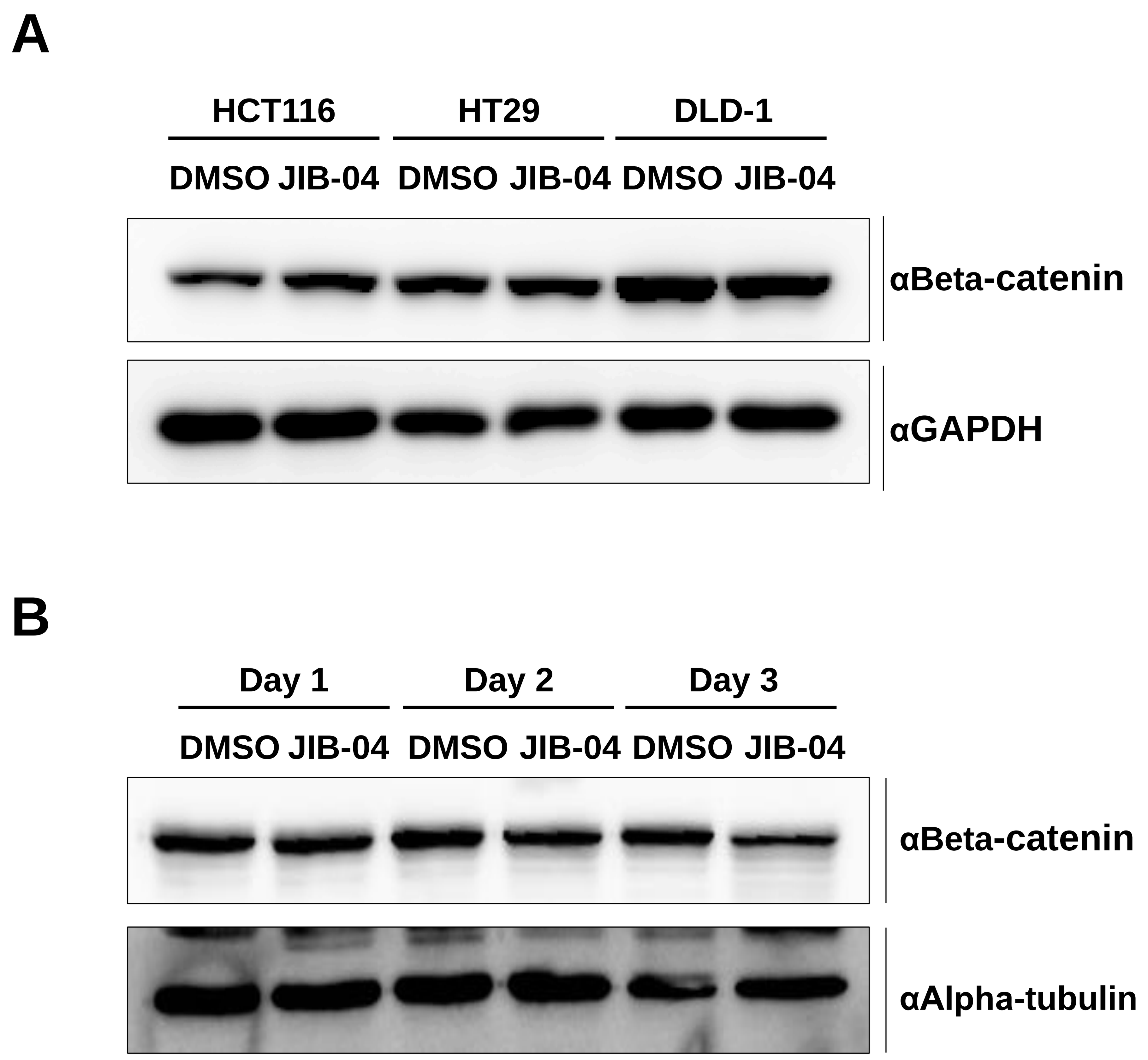

Supplementary Figure S4. A) Protein expression of β-catenin after treatment with DMSO or 2μM JIB-04 for 24h in three CRC cell lines. GAPDH was used as a loading control. B) Protein expression of β-catenin in HCT116 cells after treatment with DMSO or 2μM JIB-04 for 24h, 48h, and 72h. Alpha-tubuilin was used as a loading control.

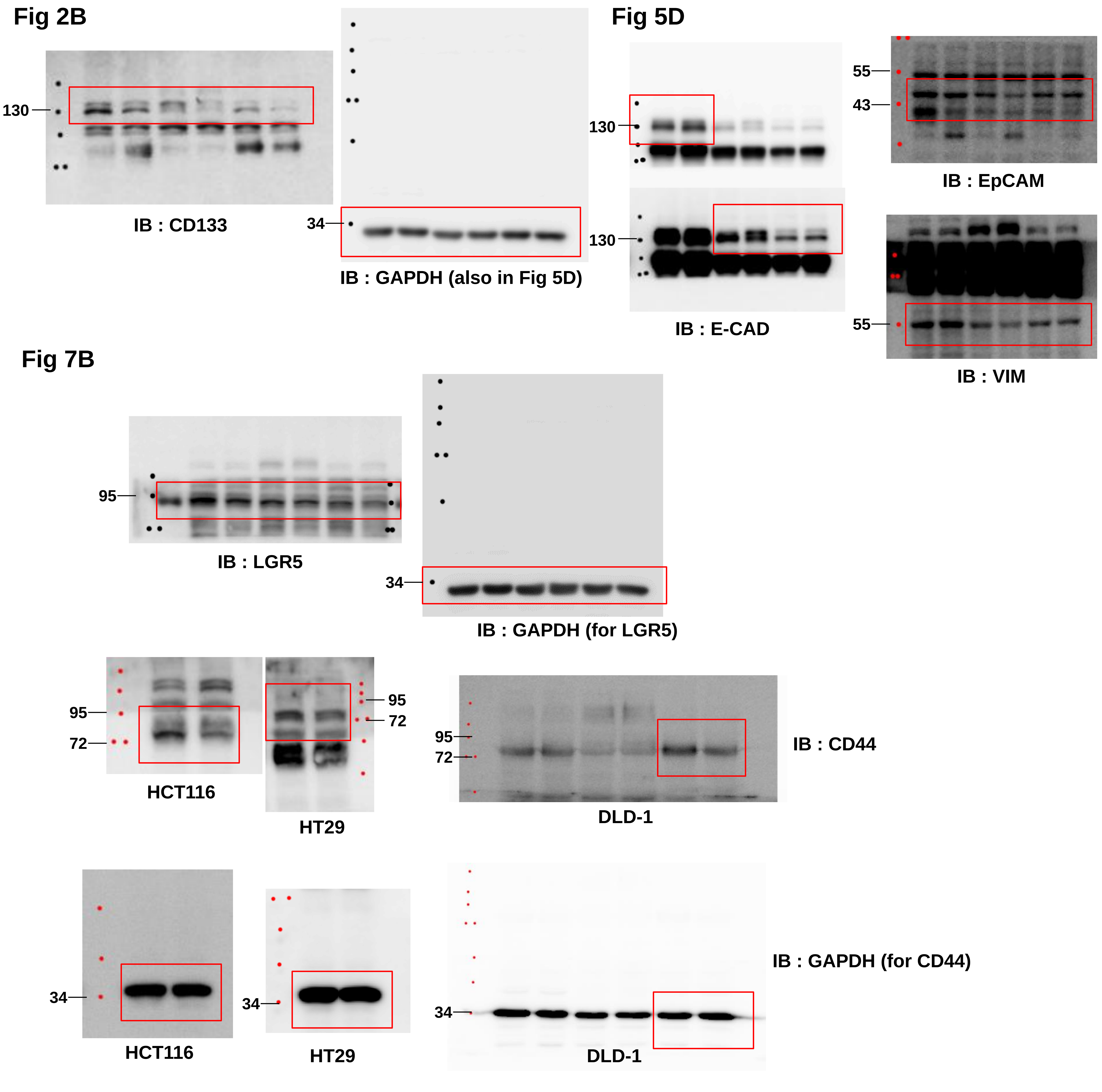

Supplementary Figure S5: Uncropped gel image from Western blot: Fig 2B, Fig 5D and Fig 7B.

Fig 7D

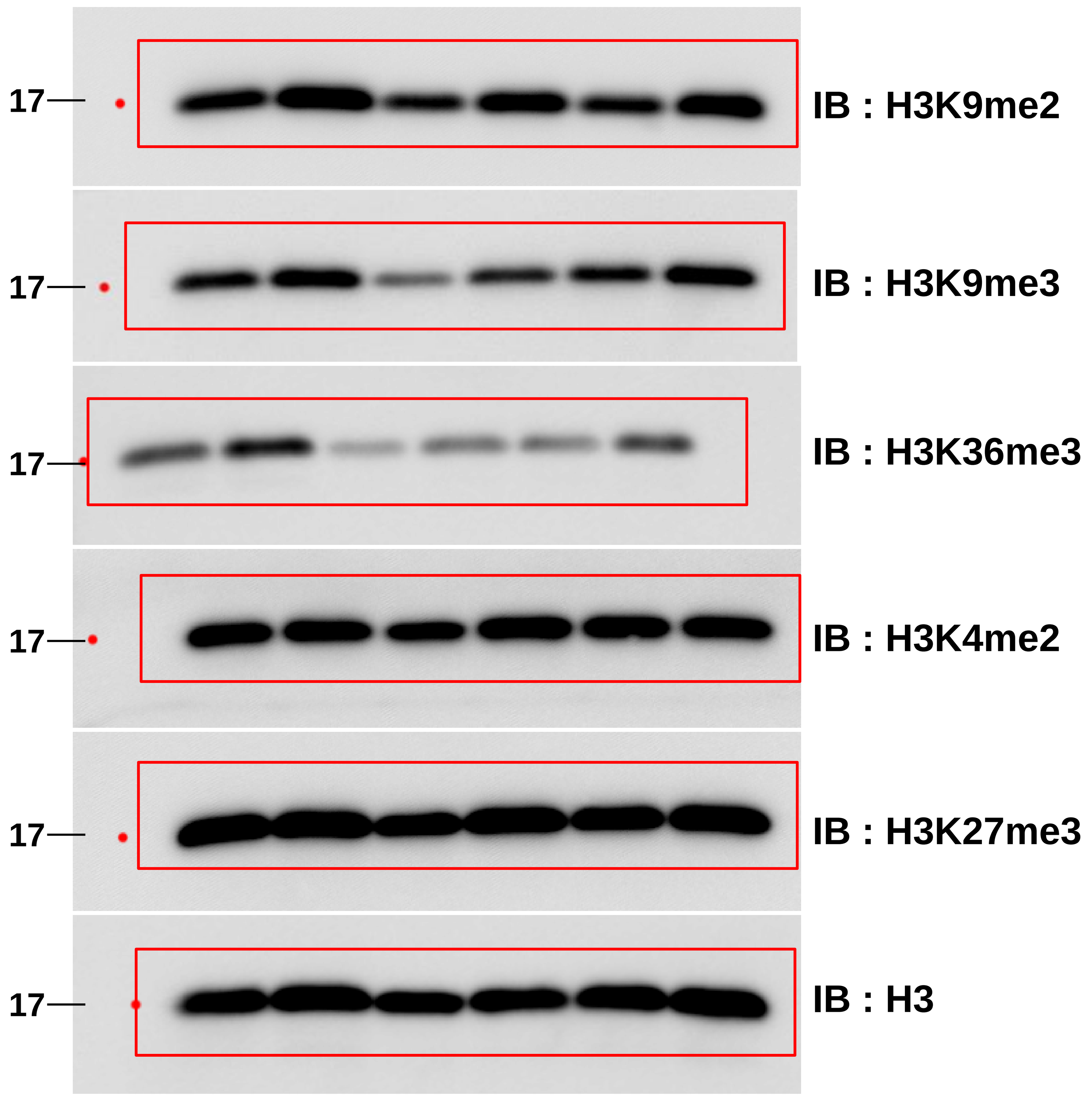

Fig S4A

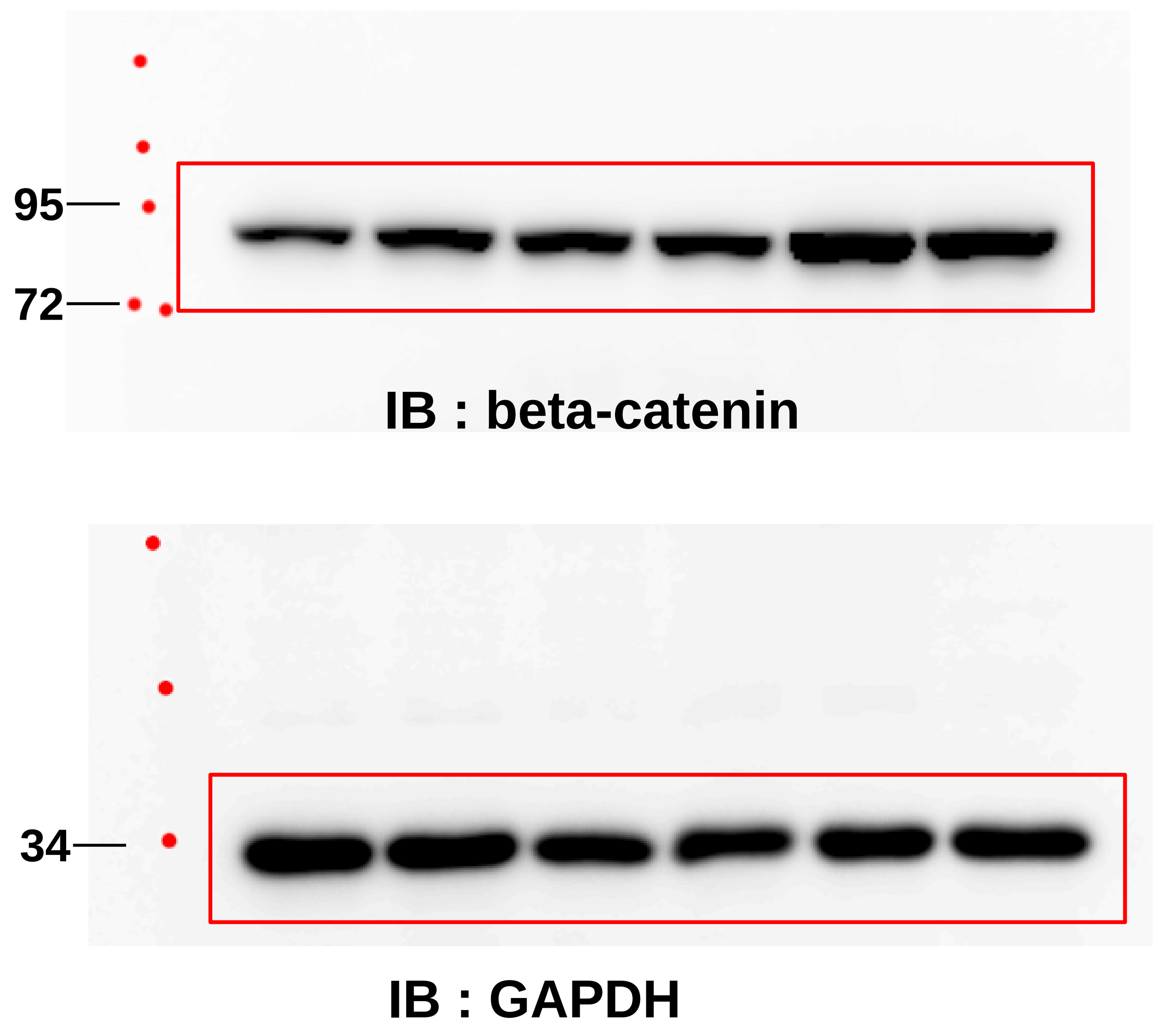

Fig S4B

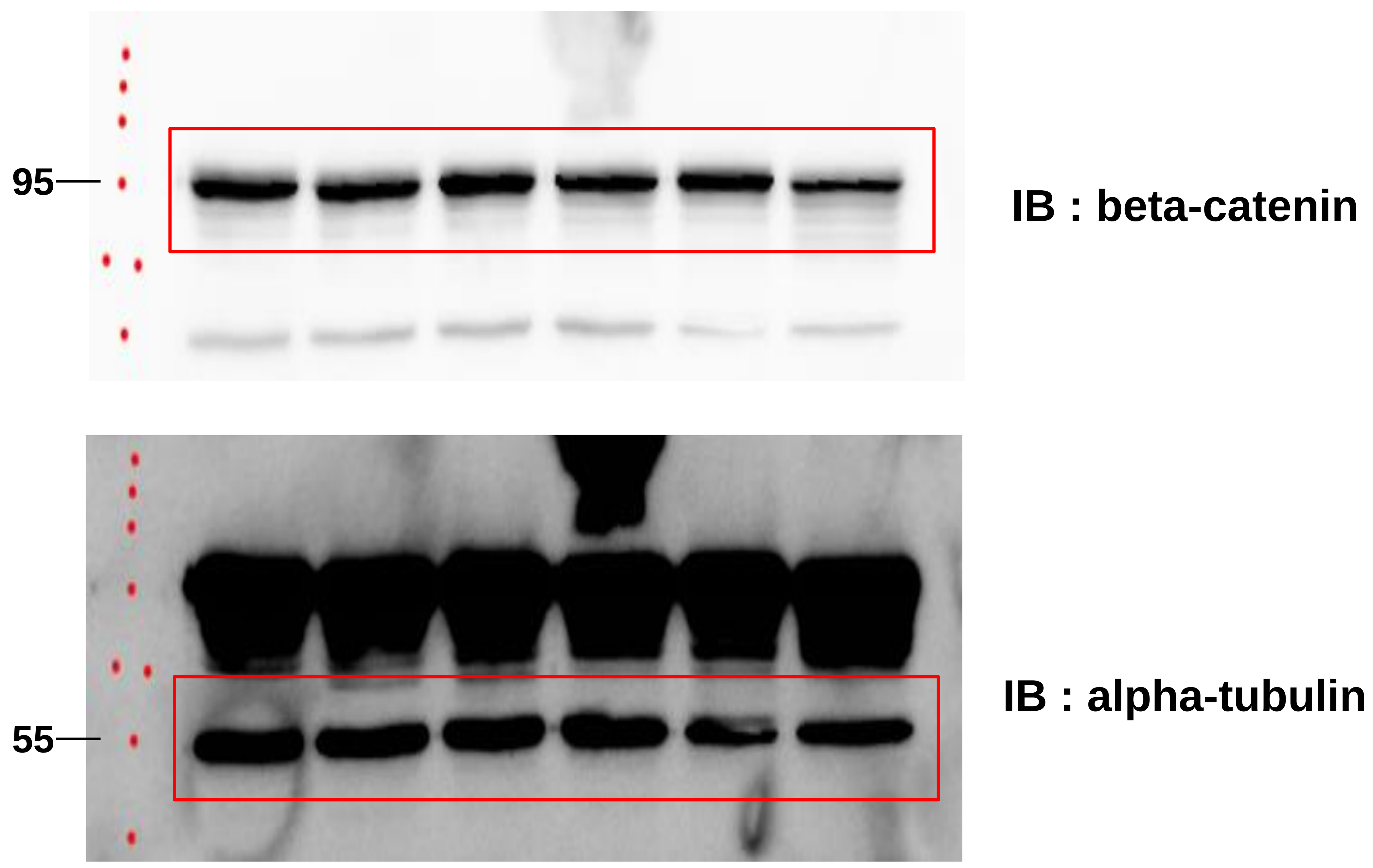

Supplementary Figure S6: Uncropped gel image from Western blot: Fig 7D, Fig S4A and Fig S4B.

Supplementary Table S1. Primer Sequences used for qRT-PCR

	Forward	Reverse
CD133	tgcagtggatcgagttct	Tcctatgccaaaccaaaa
E-CAD	aggaggcggagaagagga	gagggttggtgcaacgtc
VIMENTIN	gagaactttgccgttgaagc	tccagcagcttcctgtaggt
N-CAD	gccttaaagcggctgaca	gagctcaaggacccagca
CD44	gggggtcccataccactc	ccaagagggatgccaaga
LGR5	cctgtccttgcctgtgct	ccaccctgagcaacatcc
HIF1a	ggcagcaacgacacagaa	ttgagtgcagggtcagca
DKK1	catcagactgtgcctcagga	tatccggcaagacagacctt
ALDH1A3	tctcgacaaagccctgaagt	ggccaaagcgtattcaccta
ALDH1B1	agagggccaacaacacca	ccctcccgtttccagatt
CD24	cccacgcagatttattccag	accacgaagagactggctgt
SOX4	aagtaccggcccaggaag	ctgccaccgaccttgtct
GAPDH	tgatgacatcaagaaggtggtgaag	tccttggaggccatgtgggccat
